# Disinfection of Ocular Cells and Tissues by Atmospheric-Pressure Cold Plasma

**DOI:** 10.1371/journal.pone.0033245

**Published:** 2012-03-14

**Authors:** Paola Brun, Paola Brun, Maria Vono, Paola Venier, Elena Tarricone, Velika Deligianni, Emilio Martines, Matteo Zuin, Silvia Spagnolo, Roberto Cavazzana, Romilda Cardin, Ignazio Castagliuolo, Alvise La Gloria Valerio, Andrea Leonardi

**Affiliations:** 1 Histology Unit, Department of Biomedical Sciences, University of Padua, Padua, Italy; 2 Department of Molecular Medicine, University of Padua, Padua, Italy; 3 Department of Biology, University of Padua, Padua, Italy; 4 Department of Ophthalmology, S. Antonio Hospital, Padua, Italy; 5 Consorzio RFX, Euratom-ENEA Association, Padua, Italy; 6 Department of Neuroscience, University of Padua, Padua, Italy; 7 Department of Surgical Oncological and Gastroenterological Sciences, University of Padua, Padua, Italy; University of Illinois at Urbana-Champaign, United States of America

## Abstract

**Background:**

Low temperature plasmas have been proposed in medicine as agents for tissue disinfection and have received increasing attention due to the frequency of bacterial resistance to antibiotics. This study explored whether atmospheric-pressure cold plasma (APCP) generated by a new portable device that ionizes a flow of helium gas can inactivate ocular pathogens without causing significant tissue damage.

**Methodology/Principal Findings:**

We tested the APCP effects on cultured *Pseudomonas aeruginosa*, *Escherichia coli*, *Staphylococcus aureus*, *Candida albicans*, *Aspergillus fumigatus* and *Herpes simplex* virus-1, ocular cells (conjunctival fibroblasts and keratocytes) and ex-vivo corneas. Exposure to APCP for 0.5 to 5 minutes significantly reduced microbial viability (colony-forming units) but not human cell viability (MTT assay, FACS and Tunel analysis) or the number of HSV-1 plaque-forming units. Increased levels of intracellular reactive oxygen species (ROS) in exposed microorganisms and cells were found using a FACS-activated 2′,7′-dichlorofluorescein diacetate probe. Immunoassays demonstrated no induction of thymine dimers in cell cultures and corneal tissues. A transient increased expression of 8-OHdG, genes and proteins related to oxidative stress (OGG1, GPX, NFE2L2), was determined in ocular cells and corneas by HPLC, qRT-PCR and Western blot analysis.

**Conclusions:**

A short application of APCP appears to be an efficient and rapid ocular disinfectant for bacteria and fungi without significant damage on ocular cells and tissues, although the treatment of conjunctival fibroblasts and keratocytes caused a time-restricted generation of intracellular ROS and oxidative stress-related responses.

## Introduction

A plasma is an ionized gas in which a fraction of the atoms or molecules are ionized. The plasma is composed of electrons, ions and neutral particles [1]. Typically, plasmas produced in the laboratory contain electrons that have a very high temperature, approximately 1 eV or 11,600 K, and ions and background gas that have a much lower, close to room temperature, for which they are described as “low-temperature” or “cold” plasmas. The high electron temperature induces a wealth of chemical reactions, driving the production of chemically active species, such as free radicals and excited molecules. The use of plasmas for the treatment of living tissue has attracted attention only in recent years, following the refinement of techniques for the production of stable plasmas at atmospheric pressure, a condition essential for *in vivo* applications [2]. In particular, the development of plasma sources operating at low power levels (few Watts at most) allowed for obtaining plasmas exceptionally suited for treating sensitive targets, including living cells and tissues, without causing thermal damage [3], [4]. Recent studies have provided evidence for using the properties of low power plasma produced at atmospheric pressure to interact in a non-destructive way with living tissues [5]–[7]. In fact, plasma density and composition can be varied to control the active chemical species produced within the plasma itself. It has been demonstrated that plasma can be applied to the skin without any unpleasant feeling [8], [9].

The ocular surface is continuously exposed to microorganisms that can cause or aggravate infections like bacterial conjunctivitis and keratitis. In particular, the latter is considered an ocular emergency that requires immediate and appropriate treatment to limit corneal morbidity and vision loss. Certain microbes such as *Staphylococcus spp* and *Pseudomonas aeruginosa* are more likely to cause bacterial keratitis and, of all possible fungal infections, the vast majority is caused by *Aspergillus* and *Candida* [10]–[13]. Initial therapy for keratitis involves topical fluoroquinolones or topical broad-spectrum, fortified antibiotics. However, the emergence of bacterial resistance to topical antibiotics and antifungal drugs compromises an effective immune response and is exacerbated by the need for prolonged therapeutic treatment [14]. For these reasons, novel, immediate therapeutic strategies that are irrespective of the type of infection would be a great breakthrough in treatment.

The bactericidal effects of low temperature plasma have been well demonstrated [15]–[19], and are mainly due to the action of reactive oxygen species (ROS) [20], [21]. Since ROS-induced DNA lesions could be fixed in point mutations, the effects of plasma doses on DNA need to be investigated before using them for tissue disinfection. Recent reports have indicated that plasma-induced intracellular ROS formation causes inactivation of *E. Coli* by lipid peroxidation and oxidative DNA damage [22]. Plasma doses that substantially reduce cell viability and cause apoptosis in mammalian cells also induce significant DNA damage [23], [24]. In addition to DNA lesions, oxidation of DNA precursors can also affect biological processes including mutagenesis, senescence and neurodegeneration in mammals [25]. One of the key questions to resolve before using plasma in medical treatment is whether the exposure time necessary to obtain a disinfectant action also induces critical alterations in cells and tissues.

We previously reported that application for up to 5 minutes of atmospheric pressure cold plasma (APCP) generated by ionization of a helium flow in a new portable device exerted an antimicrobial effect without any visible microscopic changes in the corneal tissue [26]. The same plasma source was used for the experiments described in the present paper. The source is made of two coaxial copper tubes, closed at one end by brass grids and separated by an insulating layer. A sinusoidal voltage at a frequency of a few MHz with a typical value of 900 V peak-to peak is applied to the inner tube, while the outer one is kept grounded. Helium gas, having a typical flow rate of 1.75 liters/minute, is injected into the inner tube and flows to the region comprised between the two grids, where it is ionized. The resulting plasma induces the formation of reactive chemical species derived from the air that mixes with the helium flow. In particular, atomic oxygen and hydroxyl radicals are among the species that are considered to be relevant for bactericidal effects. Ultraviolet radiation, also produced within the plasma, has a secondary or negligible role [27]–[28]. As the plasma is formed between the two grids, it does not come in direct contact with the substrate to be treated. The helium flow carries the reactive species with it through the outer grid.

In contrast with what has been reported elsewhere [19], the bactericidal effect of this plasma system was as strong as the direct plasma applied by the needle developed by Stoeffels and collaborators [29]. During treatment with the plasma source used in the present study, the outer-grounded electrode was not in direct contact with the substrate to be treated, but rather stood slightly above it (usually 1 or 2 mm).

The first aim of this study was to ascertain if a short APCP pulse could significantly reduce the load of typical ocular pathogens without causing critical tissue damage. In the ocular cells and tissues treated with APCP, we also analyzed the presence of *8-oxodeoxyguanosine* (8–OHdG) and the expression of OGG1, a DNA glycosylase specific for the removal of pre-mutagenic 8–OHdG lesions, glutathione peroxidase 1 and NRF2, the genes involved in the enzymatic cell response to oxidative stress [30]–[32]. We also investigated the role of the UV component of APCP in the resulting cellular reaction.

## Materials and Methods

### Source of atmospheric pressure cold plasma (APCP) and exposure mode

In this study, we used a plasma source based on the ionization of helium flow built at the Consorzio RFX in Padua, Italy. Plasma radiation was detected with a mini-spectrometer (Hamamatsu C10082 CA) integrated with a back-thinned type linear CCD image sensor (2048 pixels). The spectrometer provides measurements with a range from 200 to 830 nm in a single spectrum. A quartz optic fiber with a 1 mm diameter core, 100 cm long, matched to the spectrometer, was 2 mm in front of the plasma. The spectrum of APCP radiation obtained with a helium flow of 1.75 liters min^−1^ at room pressure displays the presence of the N_2_ molecule and high concentrations of OH radicals (309 nm), with most of the lines concentrated in the UV region, although it is not a very effective source of high-energy UV emission [26].

### Microorganisms, virus, tissues and cells

*Escherichia coli*, *Staphylococcus aureus*, *Pseudomonas aeruginosa*, *Candida albicans*, and *Asperigillus fumigatus* strains were isolated from clinical specimens of human skin, mucosa and sputum collected at the Microbiology and Virology Section, University Hospital of Padua. Isolates were cultured on agar plates and identified according to the appearance of colonies, growth conditions and metabolic enzymatic activities. *Herpes simplex* virus type 1 (HSV-1) strain 16 was propagated in Vero cells (ATCC, Manassas, VA). The viral titer was determined by standard plaque technique and expressed as plaque forming units (PFU)/ml. Aliquots of viral stock (10^8^ PFU/ml) were stored at −80°C.

*Human corneas* unsuitable for transplantation were purchased at the Veneto Eye Bank foundation (Venice, Italy). Written informed consent was obtained from donors' relatives for the use of tissues for research purposes. We also asked for approval from the local Ethics Committee at the Padua Hospital and University. The study conformed to the Declaration of Helsinki. Corneas were stored in humid chambers at 37°C and used within 4 days.

#### Keratocytes

Keratocytes were isolated from human corneas following the method of Petroll et al [33] with some modifications. The central corneal button was obtained from a normal cornea less than 3 days after harvesting. After the corneal epithelium was removed with a cell scraper, the endothelium was enzymatically detached using 0.05% trypsin/0.02% EDTA-solution (Sigma) for 15 minutes at 37°C. The stroma was then cut into 3- to 4- mm pieces and treated overnight at 37°C with type I collagenase (100 U/ml, Worthington Biochemical) and hyaluronidase (2 mg/ml, Sigma, MO, USA) solutions in Dulbecco's Modified Eagle's Medium/F12 (DMEM/F12, Lonza, Basel, Switzerland). Isolated keratocytes were then collected by centrifugation and washed two times in DMEM/F12. The remainder of the partially digested stromal pieces was also washed and maintained in tissue culture plates containing DMEM/F12 with 10% fetal bovine serum (FBS, Biochrom, Berlin, Germany) so that other cells began migrating from them within 3–5 days. Cells were immediately seeded in monolayer culture at 10,000 cells/cm^2^ density and cultured at 37°C with 5% CO_2_ in DMEM/F12 containing 10% FBS, 1% penicillin-streptomycin and 1% L-glutamine. Expanded cells were trypsinized and sub-cultured at a ratio of 1∶2.

#### Conjunctival Fibroblasts

After written informed consent was obtained and the local Ethics Committee of the Padua Hospital and University was informed, conjunctival tissues were collected from biopsies of 3 patients who underwent eyelid surgery (mean age 43±7). Cells were extracted according to a modified version of the Maquart *et al* protocol [34]. Briefly, tissues were cut into small pieces (2–3 mm^2^) and fibroblasts were isolated by collagenase type I digestion (20 U/ml, Worthington Biochemical) at 37°C for 12 hours. These cells were then cultured with DMEM medium supplemented with 10% FBS plus 2 mM L-glutamine and (100 U/ml]/[100 µg/ml) penicillin/streptomycin (cDMEM). Medium was changed twice a week and cells harvested by trypsin treatment. The fibroblast phenotype was verified by light and immunofluorescent microscopy using the monoclonal mouse antivimentin antibody (Sigma, MO, USA).

### Exposure to APCP

Microorganisms, virus, cells and tissues were exposed at room temperature in open culture plates (three replicates) to APCP from a height of 1.5 mm, with a helium flow of 1.75 liters min ^−1^, for 0.5, 1, 2 and 5 minutes.

### Determination of APCP effects on microorganisms

Microorganisms were grown in aerobic conditions at 37°C in Luria-Bertani medium (LB, Sacco, Como, Italy) for *E. coli* and *S. aureus*, or dispersed in brain heart infusion medium (BHI, Sacco, Italy) for *C. albicans* and *A. fumigatus*, until an OD_600_ of 0.6 (∼1×10^8^ CFU/ml) was obtained. Cultures were diluted to 1×10^4^ microorganisms/ml, centrifuged for 3 minutes at 2,000 rpm and washed twice in sterile phosphate buffer saline (PBS). HSV-1 stock aliquots were diluted to 1×10^5^ PFU/ml and washed twice by centrifugation (15,000 rpm, 15 minutes). Finally, preparations of microorganisms and virus were suspended in 200 µl of sterile fresh growth medium and placed in 24-well tissue culture plates for APCP exposure, as described above.

The inactivation of microorganisms and virus by APCP treatment was assessed by colony and plaque counting, respectively. The microorganisms untreated or treated with APCP for different times were aseptically diluted in warm PBS. Bacterial samples were cultured on agar plates and incubated at 37°C for at least 24 hours, whereas yeasts were cultured in Sabouraud agar plates and incubated at 30°C for at least 48 hours. The plates with no growth were incubated for up to 72 hours. Plates were then analyzed for the presence of colonies and growth was expressed as colony forming units (CFU)/ml. The viral suspensions untreated or treated with APCP were spread on Vero cell monolayers and incubated for 48 hours at 37°C in a humidified incubator. HSV-1 infection was assessed by the presence of characteristic cytotoxic effects on eukaryotic cells and expressed as PFU/ml.

Human corneal explants were secured to polystyrene supports, kept wet in sterile PBS and scarred using a needle. Ten µl of microbial cultures (1×10^4^ CFU/ml) were deposited onto the wound and tissues were incubated for 16 hours at 37°C to allow for bacterial growth and colonization. Infected and non-infected human corneas were then positioned 1.5 mm from the tip of the plasma source and exposed for 2 minutes to APCP. After treatment, the wounded areas were gently stroked using a sterile swab. Inocula were diluted in 100 µl LB medium, plated out and incubated, as described above to estimate surviving bacterial colonies. Finally, corneas were dissected, samples were snap frozen and stored at −80°C for RNA and protein extraction, or immediately soaked in formalin and processed for apoptosis detection.

### Evaluation of the effects of APCP exposure on keratocytes and conjunctival fibroblasts

APCP effects in terms of cell viability and apoptosis rates were assessed on the third subculture passage of keratocytes and conjunctival fibroblasts explanted from three different donors.

*MTT analysis*. Cells were seeded in multi-well dishes in serum-free DMEM/F12, and covered with 200 µl of medium. They were treated with APCP for increasing time periods, then re-incubated with fresh medium until assessment of viability after 1 and 24 hours, by the MTT test (3–4,5-dimethylthiazol-2-yl-2,5-diphenyl tetrazolium bromide, Sigma, MO, USA) using a modified Denizot method [35]. With this procedure, only viable cells with functioning mitochondria can oxidize MTT to a violet-red reaction product.

#### Detection of intracellular ROS generation

Generation of intracellular ROS following plasma treatments was measured in microorganisms and human cells using 2′,7′-dichlorodihydrofluorescein diacetate (H_2_DCFDA; Molecular Probes, Invitrogen, CA, USA), a non-fluorescent probe that is rapidly oxidized to the fluorescent 2′,7′-dichlorofluorescein in the presence of intracellular ROS. Briefly, 5×10^4^ cells, *E. coli*, *S. aureus* and *P. aeruginosa* were loaded for 30 minutes at 37°C with 10 µM H_2_DCFDA in warm PBS, washed twice and placed in 200 µl of medium. Cultures were then treated for 2 minutes with APCP, as previously described. At various time points (5, 30, 60 minutes and 12 and 24 hours), fluorescence intensity was determined in microorganisms and in trypsin-harvested human cells using flow cytometry (BD FACSCalibur). Since the fluorescent 2′,7′-dichlorofluorescein rapidly dissipates from yeast, 1×10^4^ CFU/ml *C. albicans* and *A. fumigatus* were suspended in 50 mM sodium phosphate buffer containing 10 µM H_2_DCFDA (stock solution 1 mM in ethanol) and incubated for 15 minutes at 28°C [36]. Colony counting performed after staining revealed a viability of yeasts greater than 97%. Yeasts were suspended in 200 µl of medium and treated with APCP. At fixed time points (5, 30, 60 minutes, 12 and 24 hours), fluorescence was measured in a Hitachi F2000 spectrofluorimeter at excitation and emission wavelengths of 485 and 530 nm, respectively.

In some experiments, H_2_DCFDA-loaded microorganisms and human cells were treated with 5 mM *N*-acetyl L-cysteine (NAC, Sigma, MO, USA) for 15 minutes at 37°C and then exposed to APCP. To verify the effects of NAC supplementation on microorganisms, growth kinetics were determined for each bacterial and yeast strain, by monitoring the OD_600_ during a 24-hour incubation period.

#### Analysis of Cell Cycle Distribution

Keratocytes and conjunctival fibroblasts were exposed for 2 minutes to APCP and then cultured under optimal conditions for 2–24 hours. Cells were detached by trypsin digestion, washed twice in PBS, and fixed in 70% cold ethanol (30 minutes at −20°C). Cells were then washed once in citrate phosphate buffer (0.2 N Na_2_HPO_4_ and 0.1 M citric acid, 24∶1), then in PBS and finally incubated in an RNAse solution (100 µg/mL in PBS). After 30 minutes at 37°C, cells were incubated in a propidium iodide solution (PI, Sigma, 100 µg/mL in PBS) at room temperature for a further 30 minutes. DNA fluorescence was analyzed on a BD FACSCalibur flow cytometer (Becton Dickinson, New York, USA). Results were processed with the WinMDI 2.9 (Windows Multiple Document Interface for Flow Cytometry) program. For cell cycle analysis, data were expressed as fractions of cells in different cycle phases. To evaluate the effects of APCP-induced ROS on cell viability, in some experiments human cells were treated with 5 mM NAC for 15 minutes at 37°C and then exposed to APCP.

#### Externalization of phosphatidylserine

Surface exposure of phosphatidylserine (PS) by apoptotic cells was assessed in human cells pretreated or not with NAC at 2, 6 and 12 hours after APCP exposure. Cells were detached by trypsin, washed by centrifugation (1600 rpm, 8 min) and incubated for 15 minutes in the dark with Annexin V-FITC and propidium iodide solution, according to the manufacturer's instructions (CytoGLO AnnexinV-FITC Apoptosis Detection kit; Histo-line Laboratories, Milan, Italy). Samples were then analyzed using the BD FACSCalibur flow cytometer.

#### Tunel assay

1×10^5^ cells were seeded onto 22×22 mm coverslips in multiwell culture dishes and exposed to plasma for up to 5 minutes. Apoptotic cells were identified after 24, 48 and 72 hours and after 6 days, using an “In Situ Cell Death Detection” kit (Promega, WI, USA) following the manufacturer's instructions. Briefly, cell monolayers were fixed in 4% formaldehyde and permeabilized with 0.2% Triton ×100. Fluorescein deoxynucleotides were determined in the presence of the enzyme, terminal deoxynucleotidyl transferase (TdT). Coverslips were counterstained with Hoechst (Sigma, MO, USA) to mark all nuclei and fluorescein-12-dUTP-labeled nuclei were determined in randomly selected fields using a fluorescence Zeiss Axioplan microscope (Germany) equipped with a digital camera.

### Analysis of APCP-treated corneal tissues

The effects of exposure of ex-vivo corneas to APCP were evaluated in terms of morphological changes, apoptosis and induction of thymine dimers (see below). Corneas were placed in polystyrene dishes, kept wet in sterile PBS, treated with plasma for up to 5 minutes and then embedded in paraffin. Tissue sections of 5 µm were obtained. Corneal morphology was evaluated by hematoxylin and eosin staining and compared to control tissues.

Apoptotic cells in treated corneal tissue were identified with the ApopTag Peroxidase In Situ Apoptosis Detection Kit (Millipore, MA, USA). Briefly, paraffin-embedded tissues were de-paraffinized and proteins were digested by applying proteinase K (Promega, WI, USA, 20 µg/mL) to specimens. Endogenous peroxidase was quenched in 3% hydrogen peroxide in methanol for 5 minutes. As described by the manufacturer, DNA strand breaks were labeled by attaching them to biotin-labeled and unlabeled deoxynucleotides. Biotinylated nucleotides were detected using a streptavidin-horseradish peroxidase conjugate. The immunoreaction product was visualized using 3,3′-diaminobenzidine tetrahydrochloride as a chromogen and Hoechst 33528 (Sigma, MO, USA) to mark all nuclei. The numbers of total and Tunel-positive nuclei were determined in randomly selected fields using a Zeiss Axioplan microscope (Germany) equipped with a digital camera.

### Analysis of DNA damage induced by APCP-produced ROS

#### Thymine dimer (TD) localization in the nuclei of corneal tissue

The formation of pyrimidine dimers (thymine or cytosine dimers or thymine-cytosine heterodimers) consequent to UV irradiation can impair DNA replication and transcription. We evaluated the presence of thymine dimers (TD) in the nuclei of APCP-exposed cells using the system developed by Al-Adhami *et al.*, with some modifications [37]. Briefly, corneal specimens treated with APCP for up to 5 minutes were included in optimum cutting temperature (OCT) and frozen at −80°C. At the time of analysis, tissue sections were fixed with 4% formalin and treated with 0.2% Triton X-100 and 5% BSA for 1 hour at room temperature. Tissues were then washed in PBS, treated with proteinase K (20 µg/ml; Promega, WI USA) for 15 minutes and then incubated with a mouse anti-TD mAb (Kamiya, Biochemical Co, WA, USA) for 45 min at 37°C and subsequently overnight at 4°C. Slides were washed three times, incubated with anti-mouse rodamine-labeled antibody (Vector Laboratories) for 1 hour at 37°C and counterstained with Hoechst (Sigma, MO, USA). Positive controls were prepared by exposing the tissue sections to 8 J/cm^2^ UV rays of 254 nm for 10 minutes; negative controls were slides not incubated with the anti-TD antibody. All slides were analyzed by fluorescence microscopy (Zeiss, Germany).

#### HPLC analysis of 8-oxodeoxyguanosine

Samples of 5×10^6^ keratocytes were seeded in multi-well dishes in serum-free DMEM/F12, covered with 200 µl of medium and exposed to APCP for 2 or 5 minutes. DNA was purified at 2, 6 and 24 hours using a Wizard Genomic DNA Purification Kit (Promega Italia, Milano, Italy), and the assay for quantification of 8-OHdG adduct performed in 2 steps:

- Hydrolysis of DNA by nuclease P1 and alkaline phosphatase;
- 8-OHdG determination using HPLC-ED, which is a highly sensitive method with a detection limit of approximately 2 adducts/10^5^ deoxyguanosine (dG). Following nuclease P1 and alkaline phosphatase hydrolysis, samples were filtered through 0.22-µm nylon filters (Scientific Resources, Alfatech, Genova, Italy), and 20 µl DNA per sample were injected in the HPLC (Alliance 2695, Waters, Milan). Oxidated 8-OHdG and normal deoxynucleosides were separated in a 3-mm Supelcosil LC-18-DB analytical column (7.5 cm×4.6 mm, Supelco, Bellefonte, PA) equipped with a 5-mm SupelguardTM LC-18-DB guard column cartridge.

The solvent system consisted of an isocratic mixture of 90% of 50 mM potassium phosphate, pH 5.5, and 10% methanol at a 1 ml/min flow rate.

8-OHdG was detected using an electrochemical detector (ESA Coulochem II 5200 A, Bedford, MA) equipped with a high-sensitivity analytical cell, model 5011, with the oxidation potentials of electrodes 1 and 2 adjusted to 0.15 and 0.35 V, respectively. The levels of 8-OHdG were based on the amount of dG detected in the same sample by UV absorbence at 254 nm. The 8-OHdG levels were expressed as the number of 8-OHdG adducts per 10^5^ dG. An 8-OHdG standard (Sigma-Aldrich, St. Louis, MO, USA), prepared immediately before the assay, was injected before any set of samples. The coefficient of variation was <10%; the amount of DNA required for the assay was 100 µg. Samples with lower amounts of DNA were rejected, since the risk of methodological error is acceptable only above this cut-off. The experiment was repeated three times.

#### RNA isolation and quantitative RT-PCR analysis

Keratocytes and corneal tissues exposed to APCP for 2 minutes and subsequently incubated in complete medium were sampled at different time intervals and total RNA purified using the SV Total RNA Isolation System kit (Promega, WI, USA) following the manufacturer's instructions. Two µg of total RNA were retro-transcribed using oligo random hexamers and MuLV reverse transcriptase (Applied Biosystems, Milan, Italy) to generate cDNA. Gene expression levels of nuclear factor (erythroid-derived 2)-like 2 (NFE2L2), glutathione peroxidase 1 (GPX1) and the glycosylase OGG1 were evaluated with the Real Time ABI Prism 7700 Sequence Detection System (Applied Biosystems) using the TaqMan qPCR Master Mix (Applied Biosystems) and probes from the Universal Probe Library system (UPL, Roche Applied Science, Monza, Italy). Expression of target genes was normalized to the endogenous levels of glyceraldehyde-3-phosphate dehydrogenase (GAPDH). Gene quantification was carried out using a standard curve generated by amplification of 10-fold serial dilutions of the corresponding cDNA subcloned into the pGEM-T Vector System (Promega, WI, USA). Genes, conditions of RT-PCR, primers and probes used are listed in Table 1.

**Table 1:** Genes, related primer sequences, UPL probes and annealing temperatures relative to amplicons evaluated by quantitative RT-PCR.

Gene (accession n)	Primer Sequences	UPL probe	T (°C)
GADPH (NM_002046)	fw: 5′-cgggaagcccatcacca-3′ rv: 5′-ccggcctcaccccatt-3′	60	60
NFE2L2 (NM_006164.3)	fw: 5′-acacggtccacagctcatc-3′ rv: 5′-tgcctccaaagtatgtcaatca-3′	3	60
OGG1 (NM_016827.2)	fw: 5′-cttttggacctcggctcat-3′ rv: 5′-caaatgcattgccaaggag-3′	20	60
GPX1 (NM_000581.2)	fw: 5′-caaccagtttgggcatcag-3′ rv: 5′-gttcacctcgcacttctcg-3′	77	60

fw: forward; rv: reverse; Ta: annealing temperature.

#### Western Blotting

Keratocytes and corneal tissues exposed to APCP for 2 minutes and then cultured for 2–48 hours were washed with ice-cold PBS and treated for 45 minutes at 4°C with non-denaturing RIPA lysis buffer (1% v/v Triton X-100, 0.5% w/v deoxycholic acid, 10 mM EDTA, 1 µM leupeptin, 150 nM aprotinin, 500 µM 4-(2-aminoethyl) benzenesulfonylfluoride in PBS). Particulate material was removed by centrifugation (15,000×*g* for 10 minutes, at 4°C), supernatants were collected, and protein concentrations were determined using the bicinchoninic acid (BCA) Protein Assay Kit (Thermo Scientific, Rockford, USA). Equal amounts of proteins (20 µg) were added to the sample loading buffer (62.5 mM Tris pH 6.8, 10% v/v glycerol, 2% w/v sodium dodecyl sulphate, 5% v/v β-mercaptoethanol, and 0.1% w/v bromophenol blue) and incubated for 5 minutes at 100°C. Proteins were then fractionated through a 7% w/v SDS-polyacrylamide gel and immobilized on a PVDF membrane (BioRad, CA, USA). Non-specific binding sites were blocked by incubating PVDF membranes for 1 hour at 22°C in 5% w/v non-fat dry milk in PBS containing 0.05% v/v Tween 20 (PBS-T). The PVDF membrane was then incubated overnight at 4°C with polyclonal rabbit anti-GPX, or rabbit anti-OGG1 (2 µg/ml, Santa Cruz, CA, USA). After extensive washings in PBS-T, bound antibodies were detected by incubating the PVDF membrane with horseradish peroxidase-conjugated goat anti-rabbit antibody (2 µg/ml, Sigma). Finally, the enzymatic reaction was developed using ECL detection reagents (Santa Cruz Biotechnology, CA, USA) and photographed using a VersaDoc imaging system (Bio-Rad, CA, USA). Anti-β-actin antibody (Sigma, MO, USA) was used as a loading control.

### Statistical analyses

Data are reported as the mean ± the standard error of the mean (SE). Statistical analyses were performed using the unpaired Student's *t*-test and the one-way ANOVA test, using GraphPad Prism 3.03 (San Diego, California, USA) and Stat View Graphics (Abacus Cobcept In., Berkerly, Ca, USA). *P* values less than 0.05 were considered statistically significant.

## Results

### APCP inactivated microorganisms but not virus in an exposure time-dependent manner

**Figure 1:**
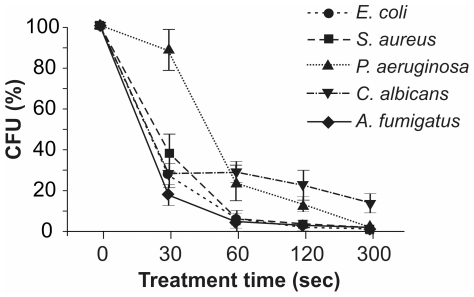
Time-dependent effects of APCP on microorganisms. Microorganisms (1×10^4^ CFU) were treated with APCP for different lengths of time, plated out and grown under optimal conditions. Microorganism survival was evaluated by the colony count assay and reported as a percentage of CFU/ml calculated in untreated samples. Data are reported as mean ± SE (error bars) of four independent experiments.

Bacterial and fungal suspensions (1×10^4^ CFU in 200 µl) were treated with APCP for 30–300 seconds, plated out and incubated under optimal growing conditions. The survival curves (percentage of colonies as a function of treatment time) reported in Figure 1 show a rapid and time-dependent inactivation of *E. coli*, *S. aureus*, *C. albicans* and *A. fumigatus*. In particular, 30 seconds of APCP treatment reduced the number of CFU/ml by 60–70%.

The calculated decimal reduction values (D value indicates time required to kill 90% of the microorganisms) were 41 seconds for *E. coli*, 38 seconds for *S. aureus* and 50 seconds for *A. fumigatus*. APCP treatment of *P. aeruginosa* caused a minor reduction of colony counts, with significant inactivation in 60 seconds and a D value of 209 seconds; Moreover, 300 seconds of APCP were not sufficient to inactivate 90% of the cultured *C. albicans* cells. After 48 hours, HSV-1 suspensions treated with APCP and cultured on Vero cell monolayer exhibited the same cytotoxic effects as those of the untreated viral suspensions in three independent experiments (data not shown).

### Effects of APCP treatment on conjunctival fibroblast and keratocyte viability

**Figure 2:**
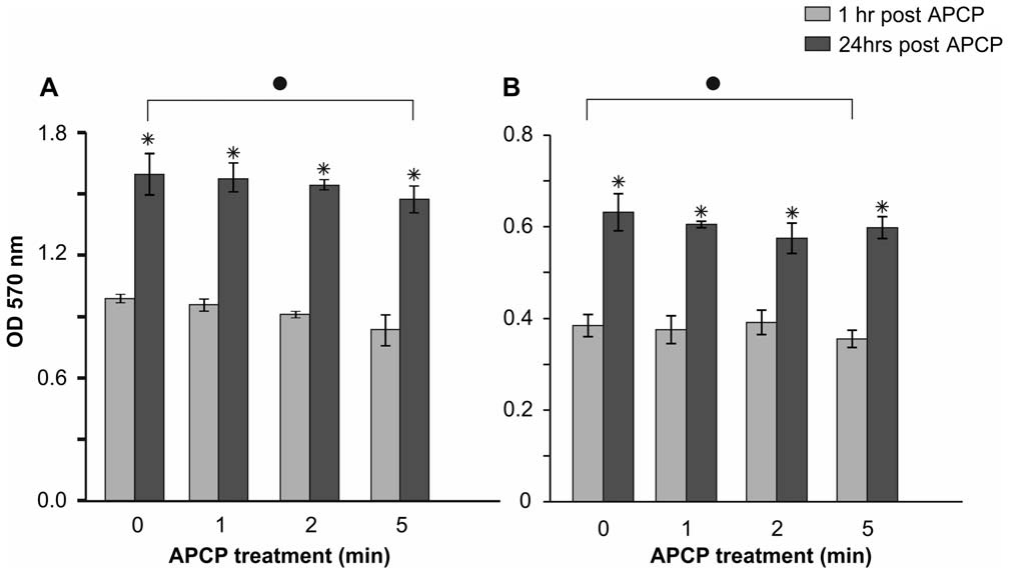
Time-dependent effects of APCP on fibroblast and keratocyte viability. (**A**) 4×10^4^ human conjunctival fibroblasts and (**B**) 5×10^4^ human keratocytes in 24-well culture dishes covered with 200 µl of medium were treated with APCP for different exposure times. The MTT test was performed 1 and 24 hours after treatment. At 1 hour, the viability of cells treated for up to 2 minutes was not reduced whereas a significant reduction of viability was found after 5 minutes of exposure (•P<0.05 compared to control values). The viability of both cells types significantly increased after 24 hours of culture (*P<0.05 compared to 1 hour of treatment). Data are reported as mean ± SE (error bars) of three independent experiments.

To determine whether APCP affected the viability of keratocytes and conjunctival fibroblasts, primary cells isolated from conjunctival or corneal tissues of three different donors were separately cultured and APCP treated for the same time intervals used with microorganisms (from 0 to 5 minutes). Cell viability was then analyzed by the MTT test. One hour after a 2-minute APCP treatment, the viability of cells was not significantly reduced when compared to untreated cells in two of the three different cell cultures. A significant reduction of viability was found in fibroblasts and keratocytes of two donors when cells were treated with APCP for 5 minutes. However, the viability of all treated cells significantly increased after 24 hours of culture (p<0.05). In Figure 2, we reported typical results relative to the cells from one donor.

Cell morphology observed with reversed optical microscopy assured that both conjunctival fibroblasts and keratocytes retained their typical morphology. Furthermore, a one-hour treatment of conjunctival fibroblasts with the same helium gas used to generate APCP did not significantly influence cell survival (data not shown).

The comparison of cell viability with that of microorganisms clearly demonstrated that 2 minutes of APCP significantly reduced the number of CFU/ml of bacterial and fungal microorganisms without changing the viability of ocular cells *in vitro*. In accordance with these findings, most of the subsequent experiments were performed with an APCP exposure time of 2 minutes.

### APCP induced ROS formation in both microorganisms and human cells

Generation of intracellular ROS following exposure to APCP for 2 minutes was evaluated in microorganisms using the H_2_DCFDA probe. Previous experiments performed in our laboratories showed that *E. coli*, *S. aureus* and *P. aeruginosa* were easily permeable to the probe, and intracellular ROS formation was evident in a dose-dependent manner following exposure to H_2_O_2_ [38]. Conversely, oxidized fluorescent H_2_DCFDA products rapidly leaked from *C. albicans* and *A. fumigates*. Based on these observations, we analyzed ROS formation using two independent experimental approaches.

As reported in Tables 2 and 3, 30 seconds after APCP exposure, we detected a significant rise in intracellular ROS levels in all bacterial and fungal strains tested. ROS levels peaked at 60–120 seconds post-APCP treatment. At this latter time point, microbial survival was also reduced (see Figure 1).

**Table 2:** APCP-induced ROS formation in bacteria.

	*E. coli*	*S. aureus*	*P. aeruginosa*
Untreated	3.81±1.46	0.41±0.08	1.52±0.31
30 sec after APCP	10.96±0.62*	32.60±1.39*	12.89±1.23*
60 sec after APCP	29.25±5.84* §	32.73±0.93*	16.94±0.34*
120 sec after APCP	33.64±6.55* §	35.25±0.53*	19.48±0.74* §

Intracellular ROS generation was measured using flow cytometry in bacteria loaded with H_2_DCFDA. Bacteria were treated with APCP for 2 minutes and analyzed at 30, 60 and 120 seconds after treatment. Data are expressed as the mean ± SE (percentage of fluorescence) of at least three independent experiments.

* *p*<0.05 *vs* untreated bacteria;

§ *p*<0.02 *vs* samples collected 30 seconds after APCP treatment.

**Table 3:** APCP-induced ROS formation in yeasts.

	*C. albicans*	*A. fumigatus*
Untreated	537±73	99±42
30 sec after APCP	727±71*	685±92*
60 sec after APCP	894±67*	1002±104*
120 sec after APCP	1078±103*	1696±140* §

Intracellular ROS formation was measured using flow cytometry in yeasts loaded with H_2_DCFDA. Samples were treated with APCP for 2 minutes and analyzed at 30, 60 and 120 seconds after treatment. Data are expressed as mean ± SE (arbitrary fluorescence units) of at least three independent experiments.

* *p*<0.05 *vs* untreated samples;

§ *p*<0.02 *vs* samples collected 30 seconds after APCP treatment.

**Figure 3:**
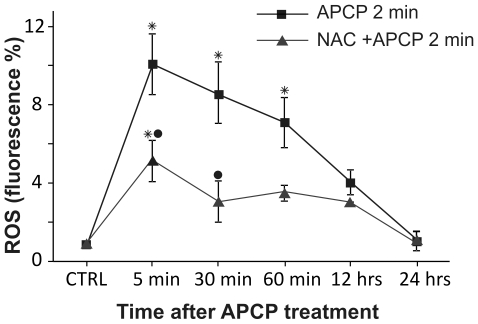
Reactive oxygen species (ROS) generated by APCP treatment generated in human cells. Primary cultures of human keratocytes were pre-treated with 5 mM *N*-acetylcystein (NAC) and exposed for 2 minutes to APCP. Cells were cultured under optimal conditions and then evaluated at different times for intracellular ROS production using the FACS-activated 2′,7′-dichlorofluorescein diacetate probe, collecting at least 10,000 events. ROS formation is expressed as percentage of fluorescent intensity. Data are reported as mean ± SE (error bars) of the percentage of fluorescence observed at least two independent experiments. (*P<0.05 compared to respective control; •P<0.05 compared to 2-minute exposure to plasma).

ROS formation was also determined in keratocytes after a 2-minute exposure to APCP. As illustrated in Figure 3, a significant peak of fluorescence was observed at 5 minutes post-treatment, with a slow return to control values within 12–24 hours. Of note, the burst of intracellular ROS was dampened by cell pretreatment with NAC. Similar results were obtained in conjunctival fibroblasts (data not shown).

### APCP induced short-term effects on human cells

**Figure 4:**
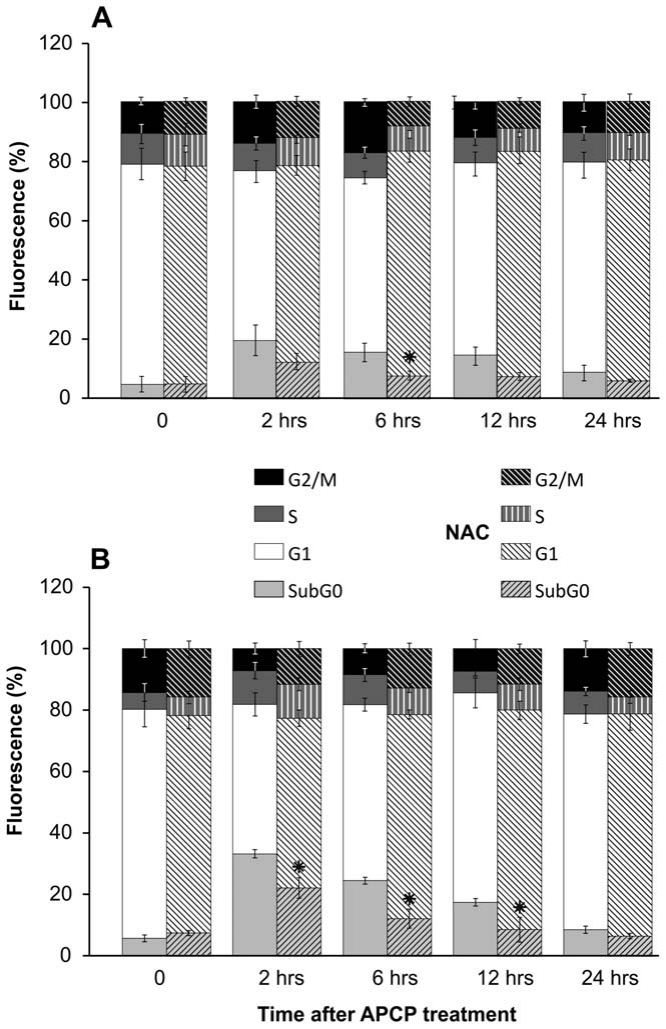
Effects of APCP treatment on human cell cycle progression. Cultured human keratocytes (panel **A**) and conjunctival fibroblasts (panel **B**) were exposed for 2 minutes to APCP with or without a 5 mM-pretreatment with NAC and subsequently cultured under optimal conditions. Cells were stained with propidium iodide and the cell cycle progression was analyzed by flow cytometry, collecting at least 10,000 events. Data are from at least two independent experiments and are expressed as mean ± SE (error bars) of percentage of fluorescence intensity in cells within each cycling phase. At 12 and 24 hours post-APCP, dead cells (sub G0) were no longer evident in samples pre-treated with NAC (*P<0.05 compared to cells exposed to APCP without NAC pretreatment). Percentages (*y* axes) indicate the fraction of cells within the drawn intervals of time (*x* axes).

To further investigate the effects of APCP-induced ROS generation in cultured conjunctival fibroblasts and keratocytes, cell cycle distribution was examined at 2–24 hours after a 2-minute exposure to APCP. According to flow cytometry, at 2 hours post-treatment, a characteristic hypodiploid peak (subG0), indicative of dead cells, was noted in keratocytes and conjunctival fibroblasts (Figure 4, panels A and B). However, dead cells were no longer evident at 24 hours post-treatment when cells showed a normal distribution of cell cycle phases. Of note, in cells pre-treated with NAC, the sub G0 peak was significantly reduced concomitantly with the ROS quencher (Figure 3).

**Figure 5:**
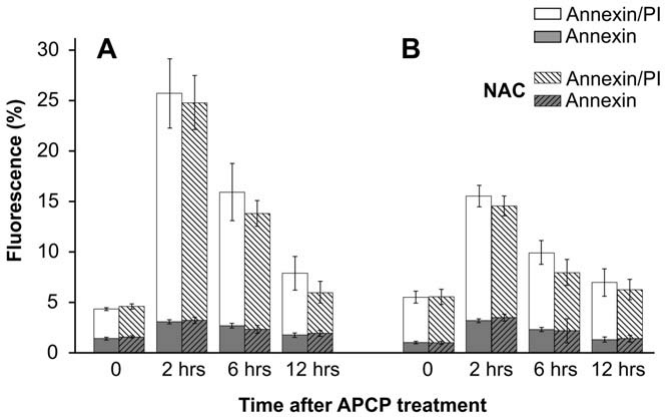
Effects of APCP treatment on externalization of phosphatidylserine. Cultured human keratocytes (panel **A**) and conjunctival fibroblasts (panel **B**) were exposed for 2 minutes to APCP with or without a 5 mM-pretreatment with NAC and subsequently cultured under optimal conditions. The externalization of phosphatidylserine was analyzed by flow cytometry after double staining of the cells with Annexin-V-FITC and propidium iodide, collecting at least 10,000 events. Single or double positive cells are expressed as a percentage of fluorescent intensity. In cells pre-treated with NAC, the increased percentage of AnnexinV/PI positive cells at 2 and 6 hours post-treatment returned to control values after 12 hours. Data are expressed as mean ± SE (error bars) of at least two independent experiments.

**Figure 6:**
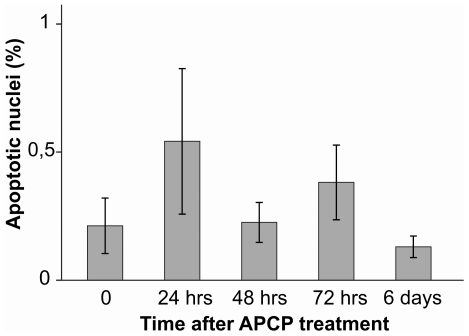
Tunel test on atmospheric pressure cold plasma (APCP) exposed keratocytes. Subconfluent keratocytes seeded onto coverslips in multiwell culture dishes were treated for 2 minutes with APCP. After 24 hours, fluorescein-12-dUTP-labeled DNA apoptotic cells were identified by fluorescence microscopy. No significant apoptotic effects were evident 24 hours after treatment. Data are expressed as mean ± SE (error bars) of at least two independent experiments.

To characterize the form of cell death, we performed a biparametric cytofluorimetric analysis using propidium iodide (PI) and AnnexinV-FITC to stain DNA and phosphatidylserine (PS) residues, respectively. Flow cytometry data indicated that 2 minutes of APCP exposure increased AnnexinV/PI positive cells at 2 and 6 hours post-treatment. However, the percentage of AnnexinV and PI-positive cells returned to control values at 12 hours post-treatment (Figure 5). This trend was confirmed by the TUNEL test on keratocyte cultures treated with APCP for 2 minutes and monitored from 24 hours to 6 days post-treatment (Figure 6). Cells pre-treated with NAC were more resistant to APCP-induced cell apoptosis (Fig. 5).

### NAC pre-treatment did not affect microorganism survival

To assess the effects of NAC supplementation on microorganism growth, each bacterial and yeast strain analyzed in this study was incubated with NAC 5 mM and grown under optimal conditions. Growth kinetics were assessed by monitoring the OD_600_ during a 24-hour incubation period. All microorganism growth was comparable to the corresponding strain not incubated with NAC, as evaluated by the time to exponential and stationary phase.

Bacterial and fungal suspensions (1×10^4^ CFU in 200 µl) were pre-treated with NAC and exposed to APCP for 2 min. After 1 min (the most effective time point after APCP exposure, see Figure 1 and Tables 2 and 3), microorganisms were plated out and incubated. Microorganism survival was evaluated by the colony count assay and reported as a percentage of CFU/ml. As detailed in Table 4, NAC supplementation did not affect bacterial and yeast survival for all microbial strains analyzed.

**Table 4:** ROS-independent effects of APCP on microorganism survival.

	*E. coli*	*S. aureus*	*P. aeruginosa*	*C. albicans*	*A. fumigatus*
60 sec after APCP	6.49±2.3	8.39±4.2	37.38±10.1	29.76±12.3	7.94±2.8
NAC treatment					
60 sec after APCP	8.07±3.0	6.62±5.2	41.13±7.6	26.18±15.1	8.58±1.3

Microorganisms (1×10^4^ CFU) were treated with APCP for 2 min with and without a pretreatment with NAC. After sixty seconds, microorganisms were plated out and grown under optimal conditions. Microorganism survival was evaluated by the colony count assay and reported as a percentage of CFU/ml calculated in untreated samples. Data are reported as the mean ± SE (error bars) of two independent experiments.

### APCP did not alter the morphology of corneal tissues

**Figure 7:**
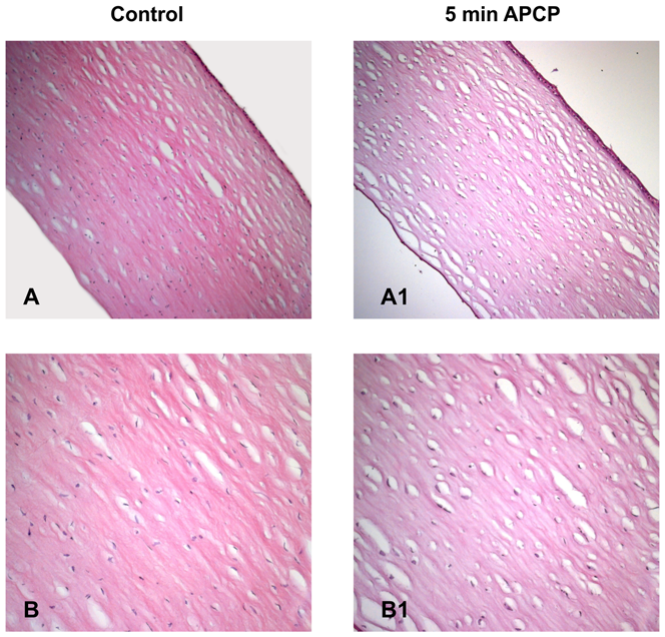
Effects of APCP treatment on ex-vivo human corneas. The tissue morphology of corneas exposed for 5 minutes to APCP was evaluated. Samples were embedded in paraffin, cut into 5 µm sections, and stained with hematoxylin and eosin. Light microscopic staining was compared to that of unexposed control tissues. A and B: control sections at 100 (A) and 200 (B) magnification; A1 and B1: treated sections at 100 (A1) and 200 (B1) magnification.

**Figure 8:**
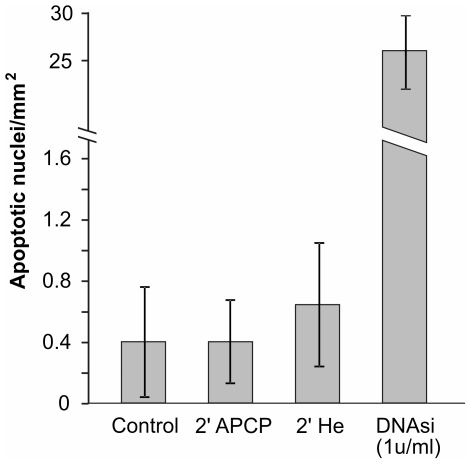
Tunel test on APCP treated corneal tissue. Apoptotic cells in 5 µm paraffin-embedded corneal tissues exposed for 2 minutes to APCP were identified by labelling DNA strand breaks with biotin-labeled deoxynucleotides and peroxidase. The immunoreaction product was visualized using 3,3′-diaminobenzidine and light microscopy. No significant apoptotic effects were evident in corneal tissues treated with APCP or only with helium (He). Data are expressed as the mean ± SE (error bars) of at least two independent experiments.

Treatment of ex-vivo human corneas with APCP for 2 minutes did not induce any change in the corneal stroma but caused a partial epithelial cell detachment (Figure 7 and Figure 8).

**Figure 9:**
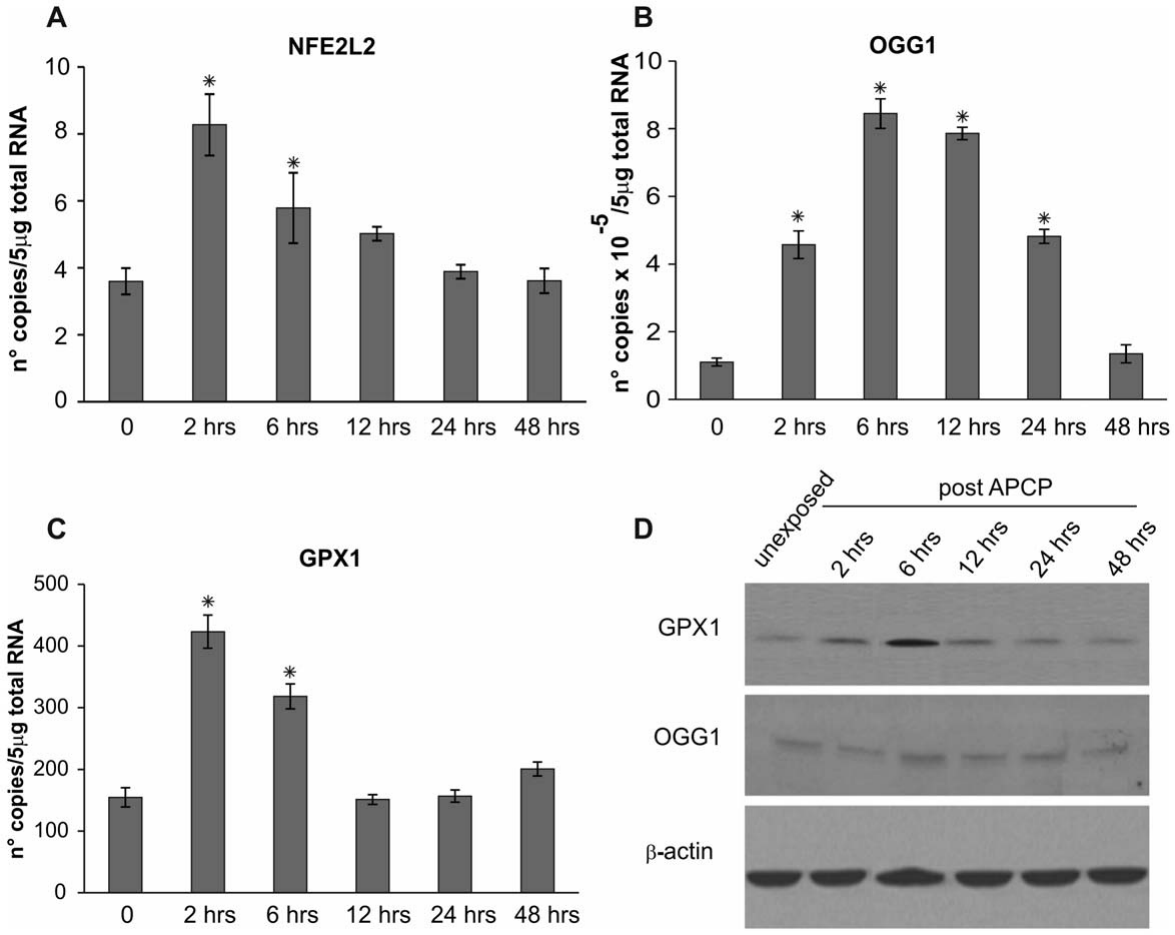
APCP-induced oxidative burst elicited short-term adaptive responses in mammalian cells. Human keratocytes were exposed for 2 minutes to APCP and cultured under optimal conditions. The mRNA transcript levels specific for human nuclear factor (erythroid-derived 2)-like 2 (NFE2L2, panel A), DNA glycosylase OGG1 (panel B) and glutathione peroxidase 1 (GPX1, panel C) were evaluated by quantitative real-time PCR. Expression of the target genes was normalized to the endogenous levels of glyceraldehyde-3-phosphate dehydrogenase (GAPDH). Data are expressed as mean ± SE (error bars) of gene copies in 5 µg of loaded total RNA, obtained from two independent experiments. ^*^ *P*<0.05 *vs* untreated cells. Protein expression of GPX and OGG1 in keratocytes was confirmed by Western blot analysis of samples taken at different time points after APCP treatment (panel D).

Evaluation of DNA fragmentation (TUNEL test) performed in parallel did not reveal significant apoptotic effects in corneal tissues (Figures 8 and Figure 9), as the number of apoptotic nuclei was comparable to those detected in control tissues. No significant apoptotic effects were evident when corneal tissues were treated only with helium.

### APCP caused transient 8-OHdG expression and adaptive responses to ROS in human cells at the transcriptional and translational level

As shown in Table 5, significant 8-OHdG levels were detected in keratocytes at 6 hours after a 5-minute exposure to APCP, while the rise observed after a 2-minute APCP treatment was not significant. However 8-OHdG amounts returned to control levels after 24 hours, the time at which the burst of intracellular ROS disappeared (see Figure 3).

**Table 5:** HPLC analysis of 8-oxodeoxyguanosine in APCP treated keratocytes.

Time after APCP treatment	2-minute APCP treatment	5-minute APCP treatment	P value
Control	16.150±1,95	10.9±3.5	
2 hrs	14.950±1.45	10.15±1.0	n.s.
6 hrs	34.650±10.35	32.250±3.75	0.05
24 hrs	13.3±1.1	17.1±1.05	n.s.

DNA was extracted from 5×10^6^ cells and the results are expressed as mean ± SE.

*p*<0.05 *vs* untreated samples.

To define the cellular responses to APCP, the expression of selected genes and proteins was evaluated in keratocytes by quantitative real-time PCR analysis and Western blotting, respectively. Expression of genes coding 8-oxoguanine DNA glycosylase (OGG1), known to remove oxidized DNA lesions, and nuclear factor (erythroid-derived 2)-like 2 (NFE2L2) and glutathione peroxidase 1 (GPX1), both involved in the adaptive response to oxidative stress, was assessed in human primary cultured cells.

Two hours after APCP treatment, OGG1, NFE2L2, and GPX1 mRNA levels significantly increased in keratocytes as compare to untreated cells (*P*<0.05, Figures 9, Panel A, B, C). Nevertheless, this increase in mRNA transcript levels was for a limited time period, reflecting the above described expression of 8-OHdG and the burst of intracellular ROS.

Western blot analysis confirmed these quantitative PCR results with regards GPX1 protein levels, which were clearly increased at 6 hours post-treatment. Following APCP treatment, the OGG1 protein levels were barely evident by Western blot analysis (Figure 9, Panel D).

### Effects of APCP on human corneas *ex vivo*

**Figure 10:**
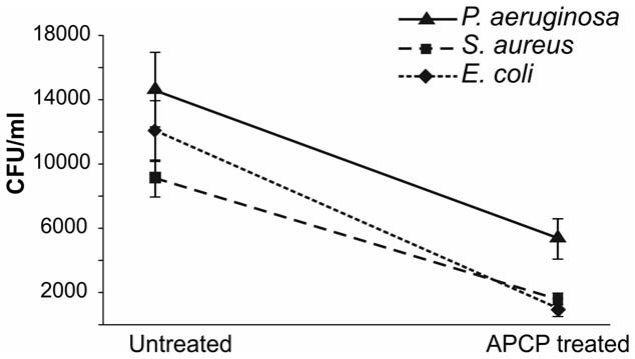
Antiseptic APCP effects on human corneas *ex vivo*. Human corneal explants were kept wet in sterile PBS. Corneas were scarred using a needle and infected with 1×10^2^ CFU bacteria and incubated for 16 hours at 37°C to allow for bacterial growth and colonization. Corneas were then exposed for 2 minutes to APCP and the wounded areas were immediately stroked using a sterile swab. Inocula were serially diluted, plated out and incubated under optimal conditions. Surviving bacteria were assessed by colony count assay and reported as CFU/ml. Data are reported as mean ± SE (error bars) of at least three independent experiments.

To validate the potential use of APCP as an antiseptic agent, we expanded the study to *ex vivo* human corneas infected with *E. coli*, *S. aureus* or *P. aeruginosa*. As displayed in Figure 10, a 2-minute treatment with APCP significantly decreased the survival of microorganisms recovered from infected tissues, thus confirming at the tissue level the disinfectant power of APCP (see Figure 1).

**Figure 11:**
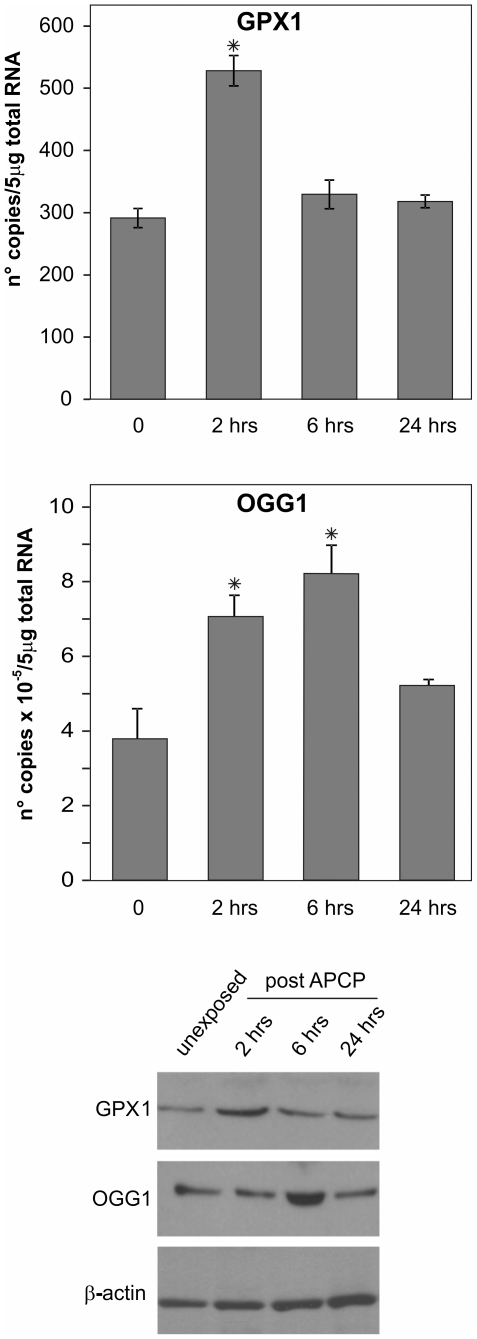
APCP treatment elicited ROS-dependent adaptive responses in ex-vivo corneas. Human corneal tissues exposed for 2 minutes to APCP were kept wet at 37°C. At different time periods after exposure, tissues were dissected and snap frozen for RNA and protein extraction. The mRNA transcript levels specific for human glutathione peroxidase 1 (GPX1, panel A) and glycosylase OGG1 (panel B) were evaluated by quantitative real-time PCR. Expression of the target genes was normalized to the endogenous levels of glyceraldehyde-3-phosphate dehydrogenase (GAPDH). Data are expressed as mean ± SE of gene copy number in 5 µg of loaded total RNA obtained from two experiments. ^*^ *P*<0.05 *vs* untreated cells. Protein expression of GPX and OGG1 was evaluated by Western blot analysis (panel C).

The effects of APCP on corneal tissues were also tested by quantitative PCR and Western blotting. GPX and OGG1 mRNA transcripts as well as protein levels rapidly increased following treatment (Figure 11), confirming in human corneas *ex vivo* both the oxidative burst and functional recovery observed in cultured corneal cells (see Figure 9 Panels A, B, C, D).

**Figure 12:**
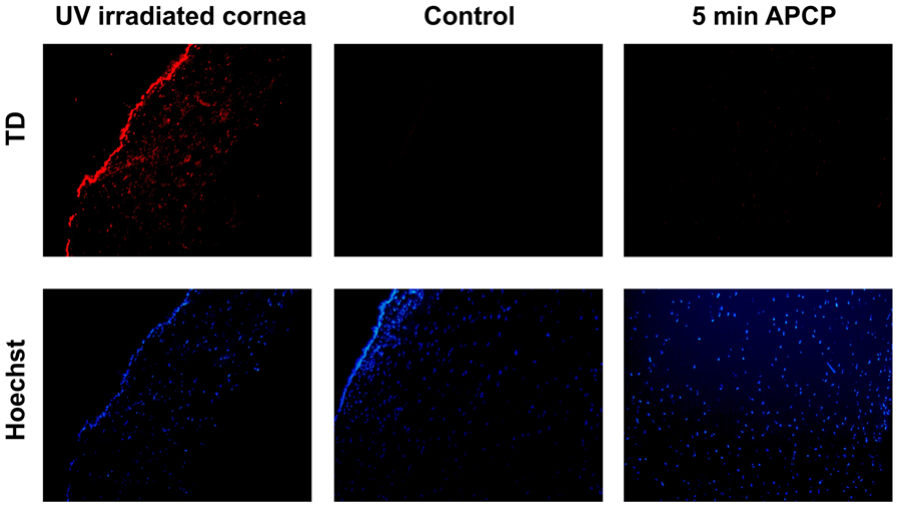
Analysis of thymine dimers (TD) in the nuclei of APCP corneal tissue. Corneal specimens exposed to APCP for 5 minutes were treated with anti-TD and counterstained with Hoechst. TD signals were not observed. Only the Hoechst staining was evident and localized in the nuclei of APCP-treated tissues;. Positive controls were prepared by exposing the tissue sections to UV rays at 254 nm for 10 minutes; negative controls were slides not incubated with the anti-TD antibody.

To ascertain if UV rays in the APCP could elicit the formation of thymine dimers (TD) at the DNA level, APCP-treated and control corneal specimens were stained with anti-TD mAb (Figure 12). Nuclei of cells exposed to APCP for up to 5 minutes did not show TD signals. In fact, anti-TD antibodies and DAPI staining co-localized in the nuclei of UV-irradiated cells, whereas only DAPI staining was evident and localized in the nuclei of APCP-treated tissues.

## Discussion

The results presented in this study indicate that a 2-minute treatment with APCP substantially inactivates ocular pathogens without causing significant tissue and DNA damage. 1×10^4^ CFU of bacterial and fungal cultures, a concentration previously demonstrated to induce infectious keratitis in rabbits [39], were exposed to APCP and after 1 minute of treatment, the number of all viable microorganisms was significantly reduced compared to controls. Both bacteria and fungi were susceptible, although the plasma treatment exerted the most pronounced bactericidal effects on *E Coli* and *S. aureus*. Such differences in effects might be due to different cell wall structures in these bacterial and fungal strains. Indeed, *P. aeruginosa* was the most resistant bacteria to the APCP exposure, showing a D value of 209 seconds. In addition to its ability to form a biofilm, which is a structure that protects these bacteria from adverse environmental factors, *P. aeruginosa* produces and secretes a variety of pigments, including pyocyanin, pyoverdine, and pyorubin, proved to scavenge ROS [40].

After exposure to APCP for 2 minutes, conjunctival fibroblasts and keratocytes maintained their viability for up to 24 hours. Reduced viability was found only in cells of some donors exposed to ACPC for 5 minutes. Flow cytometry demonstrated that after a 2-minute exposure to APCP, the transitory increase of apoptotic cells almost completely disappeared after 24 hours.

The effects of APCP treatment are not merely due to a physical effect, such as thermal load, but also to a chemical effect indirectly linked to the amount of energy dissipated from the plasma. This is particularly true for the plasma treatment used in the present study, where the plasma flow was used as a source of active chemical species without direct contact with the targeted substrate. Since the amount of active species present in the outgoing gas flow has not been precisely determined, we decided to quantify the dose through exposure time. Albeit with a different plasma source and different cell types, our data are in accordance with that of S. Kalghatgi and colleagues [23] on the effects of non-thermal atmospheric plasma, from increased cellular proliferation to apoptosis of mammalian cells. Moreover, we demonstrated that a 2 to 5-minute treatment of APCP caused intracellular ROS formation and led to microorganism death, with mild and transient effects on human cells and tissues. This evidence supports future clinical applications of cold plasma obtained with helium/oxygen, such as disinfection and antisepsis of human tissues. We also demonstrated that the treatment of human corneas *ex vivo* with 2 minutes of APCP significantly decreased bacterial colony counts without affecting the microscopic corneal structure. Hence, clinical application of APCP could be particularly useful in treating infectious keratitis, an acute ocular emergency that requires immediate and appropriate treatment to limit corneal morbidity and vision loss.

Understanding the mechanisms underlying the effects of plasma is essential to its therapeutic application. Plasmas obtained using He/O_2_ contain low UV levels [28] and we demonstrated the absence of TD formation in corneal cells and tissues treated with APCP for 5 minutes. These results are consistent with those recently reported for non-thermal atmospheric plasma [23], indicating that the UV radiation present in APCP aided in disinfection without causing genetic damage. Conversely, plasma-induced formation of ROS was definitively confirmed. After 30 seconds of APCP, levels of ROS were 3-fold higher in corneal cells than in unexposed cells. These ROS levels were significantly reduced by the oxyradical scavenger, *N*-acetyl cysteine. Under these experimental conditions, the high levels of ROS generated by a brief exposure to APCP destroyed various microorganisms, whereas the much lower, brief burst of ROS produced by exposed ocular cells did not significantly reduce the viability of corneal cells and tissues. Indeed, human cells pre-treated with NAC are protected against APCP-induced reactive species, whereas microorganism survival was unaffected. Thus, ROS produced by a 2-minute APCP treatment may not have a negative biological effect on cells per se, but the neutral plasma components and amino acid peroxidation in the extracellular medium may have a role in the activation of cellular responses to DNA damage following plasma treatment [22], [41] In particular, gatekeeper proteins like ATR (and possibly other kinases) are required to phosphorylate the H2AX histone variant and initiate pathways leading to recovery from DNA damage, apoptotic death or survival of possibly mutated cells [24]. ROS are normal metabolic byproducts that can modulate cellular processes, although excessive intracellular levels can saturate anti-oxidant and DNA repair systems and open the way to detrimental effects. Oxidized bases like *8-oxodeoxyguanosine* 8 (8-OHdG) can facilitate DNA strand breaks and act as pro-mutagenic lesions, with the cell's fate ultimately depending on a balance between physiological adjustments and irreversible changes towards cell death, senescence and degenerative processes [42]–[44]. Base oxidation of DNA, RNA and related precursors is commonly regarded as a marker of genetic damage irrespective of the endogenous or exogenous cause. We found a transient accumulation of 8-OHdG in DNA of the APCP treated cells, confirming that a 2-minute treatment with APCP significantly reduced the number of microorganisms without changing the viability of ocular cells *in vitro*. Moreover, this transient accumulation of 8-OHdG could be beneficial, stimulating cell pathways advantageous to organism health [30]. In this study, the limited non-toxic rise in ROS levels reported in human ocular cells might reflect a hormetic phenomenon defined by mild stimulatory effects at low doses and a detrimental effect at high doses [45]–[46]. To ultimately confirm the clinical value of APCP technology, the biological significance of this time-restricted ROS formation needs to be further investigated. Additional studies are in progress to establish the nature and amount of DNA damage induced by brief APCP exposure times during ocular disinfection.
